# Genetic Risk Profiles for Atherosclerosis and Venous Thromboembolism in Azorean and Mainland Portuguese Populations: A Comparative Analysis

**DOI:** 10.3390/cimb47080625

**Published:** 2025-08-06

**Authors:** Luisa Mota-Vieira, Joana Duarte, Xavier Catena, Jaime Gonzalez, Andrea Capocci, Cláudia C. Branco

**Affiliations:** 1 Molecular Genetics and Pathology Unit, Hospital of Divino Espirito Santo of Ponta Delgada EPER, Azores, 9500-782 Ponta Delgada, Portugal; luisa.mq.vieira@azores.gov.pt (L.M.-V.); joanaduarte17@gmail.com (J.D.); xavier.catena_parrado@med.lu.se (X.C.); jaimegonzalezsantander@live.com (J.G.); andrea.272@alice.it (A.C.); 2 BioISI–Biosystems & Integrative Sciences Institute, Faculty of Sciences, University of Lisboa, 1749-016 Lisbon, Portugal; 3 Molecular Medicine and Gene Therapy, Lund Stem Cell Centre, Lund University, 221 84 Lund, Sweden; 4 Wallenberg Center for Molecular Medicine, Lund University, 221 84 Lund, Sweden; 5 Asgard Therapeutics AB, Medicon Village, 223 81 Lund, Sweden

**Keywords:** Azores, Portugal, atherosclerosis, thromboembolism, genetic predisposition to disease, cardiovascular disease

## Abstract

The frequency of specific variants associated with the risk of developing cardiovascular diseases has been extensively studied through genome-wide association studies (GWASs). Differences between populations may be caused by the interaction of several factors, such as environmental and genetic backgrounds. Here, we studied 19 SNPs involved in atherosclerosis (AT) and venous thromboembolism (VTE) risk in the Azorean and mainland Portuguese populations and compared their frequencies with other European, Asian, and African populations. Results revealed that, although there was no difference between Azorean and mainland populations, eight SNPs in *ADAMTS7*, *PCSK9*, *APOE*, and *LDLR* genes showed significant statistical differences (χ^2^, *p* < 0.05) when compared with the European population. The multilocus genetic profile (MGP) analysis demonstrated that 7.4% of mainlanders and 11.2% of Azoreans have a high-risk of developing atherosclerosis. The opposite tendency was observed for venous thromboembolism risk, where the mainland population presented a higher risk (6.5%) than the Azorean population (4.1%). Significant differences in VTE-MGP distribution were found among the Azorean geographic groups (*p* < 0.05), with the Eastern group showing the highest VTE risk. Conversely, for the risk AT-MGP, the Central group shows the highest risk (12.9%). Taken together, the data suggest a risk of developing a cardiovascular disease consistent with the European population. However, the Azorean-specific genetic background and socio-cultural habits (dietary and sedentary) may explain the differences observed, validating the need to assess the allelic and genotypic frequencies between different populations, especially in small geographical locations, such as the Azores archipelago. In conclusion, these findings can improve the prevention, diagnosis, and treatment of high-risk individuals, and contribute to reducing the lifelong burden of cardiovascular diseases in the Azorean population.

## 1. Introduction

Atherosclerosis (AT) and venous thromboembolism (VTE) are important cardiovascular processes, traditionally considered two separate pathophysiological and clinical entities. Current scientific evidence on thrombus formation has proved that they share more mutual risk factors, including genetics, than previously recognized [1,2,3,4].

In recent years, hundreds of genetic loci have been identified as being associated with atherosclerosis, particularly in its manifestation as coronary artery disease (CAD) [5]. Initially identified in 2007 and subsequently validated by meta-analyses, the 9p21 locus has been established as a significant genetic risk factor for CAD, with approximately 20–25% of individuals of European ancestry being homozygous for the risk allele (~30 to 40% of the increased risk of CAD) [6,7,8]. Genes involved in lipid metabolism, like *SORT1*, *LDLR*, *PCSK9*, and *APOE*, are also implicated in CAD risk. The *SORT1* gene modulates blood low-density lipoprotein cholesterol (LDL-c) levels, by altering the secretion of very-low-density lipoprotein (VLDL) particles from the liver into the bloodstream [9,10,11]. Low-density lipoprotein receptor (LDLR) glycoprotein binds to plasma LDL particles, promoting the uptake and consecutive degradation of LDL. An alteration of the *LDLR* gene increases LDL plasma levels, which results in a higher risk of CAD [12,13,14]. Proprotein convertase subtilisin/kexin type 9 (PCSK9) and apolipoprotein E (APOE) bind to LDL receptors and also increase the LDL plasma levels in circulation [15,16,17,18]. Another perspective of atherosclerosis development consists of the genes involved in plaque formation, being the extracellular matrix protease ADAMTS-7, encoded by the *ADAMTS7* gene [19], the strongest loci associated with CAD risk.

The obstructive clot formation that defines VTE is the end product of a complex interplay between coagulation, inflammation, innate immunity, and fibrinolysis [20,21]. VTE has a genetic component where several genes are involved. Among them are factor V Leiden (*F5*), prothrombin (*F2*), and methylenetetrahydrofolate reductase (*MTHFR*). The gain-of-function mutation in *F5*, namely NM_000130.4:c.1601G>A, is responsible for the resistance to active protein C that prevents blood coagulation [22,23,24]. Individuals that carry the *F2* (NM_000506.5:c.*97G>A) variant have higher prothrombin levels, resulting in a 2.3-fold increased risk of VTE [23,25,26]. Although controversial, two variants of the *MTHFR* gene (NM_005957.5:c.665C>T and NM_005957.5:c.1286A>C) have also been associated with an increased susceptibility to develop VTE [27,28,29].

Cardiovascular diseases (CVDs) are the most relevant causes of death across Europe and cover a wide range of medical conditions that affect from the heart to the vascular system. According to Timmis et al. [30], Portugal is one of the countries with an alarming increase in crude CVD mortality, both for males and females. Also, according to the Azores Regional Health Plan 2030, in 2022, 27.3% of Azoreans died due to CVDs (Plano Regional de Saúde dos Açores 2030, public discussion document 2024 (accessed on 9 May 2025) [31]. In fact, considering diseases of the circulatory system, the Azores archipelago presented a 5.2% (698.0 per 100,000 inhabitants) decrease in years of life lost (YLL) when compared to mainland Portugal (480.2 per 100,000 inhabitants). Previous studies demonstrated that 12.4% of healthy individuals from the São Miguel Island have increased genetic risk for thrombotic events [32]; 9.1% of Azoreans are APOE4 carriers [33]; and 7.2% of the Azoreans have relevant genetic risk for CVDs [34].

In light of these considerations, we investigated the allelic and genotypic frequencies of SNPs implicated in atherosclerosis (AT) and venous thromboembolism (VTE) in two Portuguese populations: the mainland and the Azores archipelago. Moreover, we compared the genetic risk of AT and VTE between the two populations, which have different prevalence of diabetes, obesity, and arterial hypertension. Additionally, we assessed, through multilocus genetic profile analysis (MGP), whether individuals with a high-risk for atherosclerosis have an aggravated risk for a VTE event and vice versa.

## 2. Materials and Methods

### 2.1. Ethical Statement

The present study included two healthy Portuguese populations, one from the Azores archipelago and the other from the mainland. The Azorean population consists of 170 DNA samples of unrelated healthy blood donors selected from the anonymized Azorean DNA bank, located at the Hospital of Divino Espírito Santo of Ponta Delgada (HDES) [35]. This DNA bank was established after approval by the HDES Health Ethics Committee (and follows the international ethical guidelines for sample collection, processing, and storage, which include written informed consent, confidentiality, and abandonment option in the case of expressed will [36]). The previously studied mainland population [37] consists of 108 DNA samples dispersed throughout the mainland territory. Their collection also followed international ethical guidelines.

### 2.2. Study Populations

According to the last Portuguese Census (2021), the Azores archipelago have 236,413 inhabitants, representing 2.3% of Portugal’s population. In the present study, we analyzed 170 DNA samples geographically representative of the whole Azores archipelago: Eastern group—Santa Maria (n = 10) and São Miguel (n = 60); Central group—Terceira (n = 30), Graciosa (n = 10), São Jorge (n = 10), Pico (n = 10), and Faial (n = 10); and Western group—Corvo (n = 10) and Flores (n = 20). Sex representativeness was also considered, in a total of 85 males and 85 females, which were also half and half distributed in each island sample. Previous work using different sample sizes, performed by our research group [37,38], demonstrated that the Azorean population can be analyzed as a homogeneous genetic group. Simulation-based power analyses (R package genpwr) conducted across a range of sample sizes and allele frequencies, indicated that the cohort of 170 individuals would be sufficient to capture the overall genetic variability within the study population. This is especially valid considering that the majority of the selected SNPs have a minor allele frequency (MAF) above 10%, which increases the power to detect allele frequency differences with moderate effect sizes. For common variants (MAF >10%), the sample size of 170 individuals provides adequate statistical power (≥80%) to detect between-group differences with a moderate effect (Cohen’s w ~0.3) at a significance level of α = 0.05. Regarding the mainland Portuguese population, sex representativeness was not considered in the analysis, as only 7 out of 108 individuals were female. This imbalance reflects a limitation in the available data; however, only the mainland sample was accessible to the research team.

### 2.3. SNP Selection and Genotype Determination

We selected genes and SNPs from GWASs in which phenotypes were related to cardiovascular diseases. For this purpose, we gathered information (Supplementary Table S1) on population frequency of genetic variants, gene–disease associations, and gene–gene and gene–environment interactions based on several databases, including population data: dbSNP (https://www.ncbi.nlm.nih.gov/snp/; accessed on 10 April 2025), gnomAD (https://gnomad.broadinstitute.org/; accessed on 10 April 2025), and GWAS catalog (https://www.ebi.ac.uk/gwas/; accessed on 10 April 2025); genomic resource data: Ensembl (https://www.ensembl.org/index.html; accessed on 10 April 2025); pharmacogenetic data: pharmGKB (https://www.pharmgkb.org/; accessed on 10 April 2025); disease data: ClinVar (https://www.ncbi.nlm.nih.gov/clinvar/; accessed on 10 April 2025), OMIM (https://www.omim.org/; accessed on 10 April 2025), and DISGENET (https://www.disgenet.com/; accessed on 10 April 2025); and bibliographic data: PubMed (https://pubmed.ncbi.nlm.nih.gov/; accessed on 10 April 2025).

All SNPs were genotyped by TaqMan^®^ Pre-Designed SNP Genotyping Assays (Thermo Fisher Scientific, Waltham, MA, USA) in a 7500 fast real-time PCR system (Life Technologies, Waltham, MA, USA), as previously described [34].

### 2.4. Statistical Analysis

Allelic and genotypic frequencies were estimated by direct count for the mainland Portuguese and Azorean populations. These frequencies were compared with four populations from the gnomAD database (v4.1.0; https://gnomad.broadinstitute.org/; accessed on 10 April 2025), namely Europeans (non-Finish; EUR), African/African American (AFR), admixed American (AMR), and South Asian (SAS). The calculation of χ^2^ *p* values was performed using the 2-way Contingency Table Analysis webpage (http://statpages.org/ctab2x2.html, accessed on 15 June 2024). Results were considered statistically significant at *p* < 0.05. When the assumptions for the chi-square test were not met, we performed Fisher’s exact test. Haplotype analysis was computed by the Arlequin software v.3.5.1.3 (https://cmpg.unibe.ch/software/arlequin35/ accessed on 10 January 2023), through a maximum likelihood method. Hardy–Weinberg equilibrium (HWE) was determined for Azorean and mainland Portuguese populations, and no departure from HWE was observed.

To have a comprehensive view of the genetic risk for cardiovascular diseases, we performed a multilocus genetic profile (MGP) analysis in two distinct groups: (i) atherosclerosis (AT), which included *SORT1*, *ADAMTS7*, *PCSK9*, *APOE*, and *LDLR* genes, and 9p21 genomic region; and (ii) venous thromboembolism (VTE), composed of the *F5*, *F2*, and *MTHFR* genes. Based on the information about the reported phenotypic associations and the values of the odds ratio (OR) of the 19 SNPs (Supplementary Table S1), the genetic risk score (GRS), based on a weighted analysis, was computed as the sum of each risk allele multiplied by the corresponding OR, when available. To validate this approach, we additionally recalculated the MGP using the logarithm of the ORs [log(OR)], as commonly applied in polygenic risk score models to reflect additive effects on the log-odds scale. Furthermore, we performed a percentile-based analysis, where individuals were classified into genetic risk categories based on the distribution of the risk scores. Specifically, the top 10% of the distribution was defined as the high-risk group, while the bottom 10% represented the low-risk group.

## 3. Results

### 3.1. Allelic and Genotypic Frequencies

The genetic diversity in the Azorean and mainland Portuguese population results showed that rs11591147 (*PCSK9*) exhibited the lowest allele frequency (0.9%) in both Azorean and mainland populations, while rs562556 (*PCSK9*) presented the highest risk allele frequency (88.0%; Table 1). A comparison of the Azorean and mainland populations demonstrated that both had similar allelic frequencies for all SNPs, with the majority also being in agreement with those observed in the European population (χ^2^, *p* > 0.05; Supplementary Table S2). Nevertheless, *ADAMTS7* (rs3825807), *PCSK9* (rs505151), three *APOE* SNPs (rs405509, rs429358, and rs439401), and *LDLR* (rs688 and rs1433099) showed significant statistical differences (χ^2^, *p* < 0.05; even after Bonferroni correction; *p* < 0.0033) when compared with the European population, although no difference was observed between the Azorean and mainland populations. 9p21 rs10757274 was the only SNP with a significant difference, though minor, between the mainland Portuguese and the Azorean population (*p* = 0.036; 95% CI: 1.023−2.047).

### 3.2. Haplotype Structure

Haplotype diversity was estimated for the *MTHFR*, *PCSK9*, *APOE*, and *LDLR* genes, and for the 9p21 genomic region (Table 2) in the Azorean and mainland Portuguese populations. The *PCSK9* and *APOE* genes, with the same number of SNPs, exhibited similar number of haplotypes (11 vs. 12, respectively).

The most frequent haplotype in the *MTHFR* gene, with a frequency of 78.7% for the mainland and 76.5% for the Azorean population, was the H_1_−“CA” (Table 2). This haplotype, composed of non-risk alleles, showed the highest frequency in the Western group of the Azorean population. For the *PCSK9* gene, we identified 11 haplotypes, with H_7_−“**T**G**A**A” being the most frequent in both populations (0.655 in the mainland and 0.657 in the Azorean population). The H_2_−“CGGA” haplotype, which contains only non-risk alleles, showed a frequency of 1.8% in the mainland population and 3.6% in the Azorean population. For the *LDLR* gene, H_1_−“CCC” and H_2_−“CCT” were the most frequent haplotypes in the mainland and Azorean populations, respectively. Additionally, the H_8_−“TCT” was observed at a frequency of 2.0% in the mainland, but was absent from the Azorean population. In the 9p21 region, four haplotypes were identified, with H_3_−“**GC**” being the second most frequent in both the Azorean (43.4%) and the mainland population (36.9%), and showing the highest frequency in the Central group of the Azorean population (48.6%).

### 3.3. Multilocus Genetic Profiles

To evaluate genetic risk and identify high-risk individuals, we estimated multilocus genetic profiles (MGPs) by combining genotypes (SNP composition is described in Supplementary Tables S4 and S5). The AT (atherosclerosis) group results revealed 164 different MGPs for the Azorean population and 104 for the mainland Portuguese population. Percentile genetic risk analysis revealed eight MGPs for high-risk individuals in the mainland population, all with a frequency of 0.9%, and 19 MGPs in the Azorean population, with a frequency of 0.6% (Table 3). The profile with the highest genetic risk score (GRS), namely AT-MGP_1_-”G**T TT TT** GG **AA** AA G**T** TT CC C**T** CC **TT** CC **GG CC**”, was observed exclusively in the mainland population. For AT, the data showed no common genetic profiles between both populations (low- and high-risk profiles). Although not statistically significant, the Azorean population demonstrated a 1.5 times higher risk for atherosclerosis than the mainland population. At the lowest risk percentile, we did not observe statistically significant differences between the populations, neither among the sex nor the geographic groups. Nevertheless, the mainland population (12.0%) exhibited a lower risk for atherosclerosis than Azoreans (8.8%).

Considering the risk for VTE (Table 4), we observed a total of 14 different MGPs for the Azorean population and 16 for the mainland Portuese population. The profile with the highest GRS value was observed in the mainland population, with a frequency of 0.9%, VTE-MGP_1_−“G**A** G**A** C**T** AA”. The only profile common to both populations was the VTE-MGP_4_−“G**A** GG C**T** AA” with a frequency of 0.9% for mainlanders and 1.2% for Azoreans. The distribution of the genetic profiles by geographic groups showed statistically significant differences (*p* = 0.011) between the three groups, with the Eastern presenting an approximately 6 times (95% CI: 1.42–29.46) higher VTE risk than the Central group. Sex analysis revealed that women had a higher risk of VTE with a total frequency of 7.1%, a statistically significant (*p* = 0.011; 95% CI: 1.31–38.67; Bonferroni correction *p* < 0.0125) difference when compared with males (1.2%). The Western group did not present any high-risk VTE-MGPs.

Acknowledging the methodological concerns regarding the use of raw odds ratios (ORs) in the genetic risk score calculation, we conducted a validation analysis using log-transformed ORs for both atherosclerosis (AT) and venous thromboembolism (VTE) profiles. The consistency observed between the two approaches likely reflects the marginal and relatively homogeneous magnitude of the ORs used. Consequently, the classification of high-risk profiles remained unchanged, reinforcing that the conclusions are dependent on the choice of weighting method.

## 4. Discussion

The frequency and impact of risk variants associated with the risk of developing cardiovascular diseases may vary between populations due to differences in genetic background, lifestyle, and environmental exposures [6,7,8]. Here, we studied the allele frequencies of 19 SNPs in the Azorean and mainland Portuguese populations and compared them with other populations, namely Europeans (non-Finish; EUR), African/African American (AFR), admixed American (AMR), and South Asian (SAS). Results demonstrated that there are no significant differences between them. As expected, these results agree with those reported for the European population, but with differences for African, American, and Asian populations. Concerning the 9p21 region, the results evidenced that the mainland population presented, for rs133349, a smaller frequency of homozygous individuals for the risk allele (CC; 18.5%) compared to another mainland sample described by Mendonça et al. (29.3%) [39]. This may be due to the difference in sample size (683 vs. 108). Nevertheless, the results obtained were similar to those observed in the Azoreans (CC; 20.0%). For rs10757274, the data showed a significant difference, although with small effect (*p* = 0.036; 95% CI: 1.023–2.047), when we evaluated the frequency of the risk allele (G) in the Azorean and the mainland populations.

Because a haplotype is the combination of alleles in the adjacent loci that are part of the same chromosome, it may be considered an important approach for investigating the genetics of common diseases. The data demonstrated that the *PCSK9* H_8_—“**T**G**AG**” haplotype, with three risk alleles for atherosclerosis, had a similar frequency in the two populations; however, we observed that females presented a higher frequency (2.9%) than males (1.8%; *p* = 0.723). Moreover, the data showed a difference, which was not statistically significant (*p* > 0.05), in the frequency distribution between geographic groups, with the lowest value for the Central group (0.8%). On the other hand, according to the ClinVar database, the *PCSK9* SNPs are associated with familial hypercholesterolemia (FH). The early identification of FH patients can increase their life expectancy and quality of life, by preventing premature coronary heart disease, if patients receive appropriate pharmacological treatment. Taken together, these data validate the need to assess the allele, genotype, and haplotype frequencies between different populations, especially in the case of small geographical locations, such as the Azores archipelago.

Analysis of the MGPs contributes to understanding the biological basis of genetic susceptibility and the risk level in a specific population. Here, we analyzed combined alleles in two distinct groups—AT and VTE. Considering the AT risk group, we observed a high diversity of profiles for both the Azorean (164/170) and the mainland population (104/108). For the high-risk percentile, no common profile was observed between the two populations, and the Azoreans presented a higher number of high-risk MGPs. Nevertheless, 7.4% of mainlanders and 11.2% of Azoreans exhibited a high risk for atherosclerosis. The same tendency was observed in a report from the Portuguese National Institute of Statistics (2014; https://www.ine.pt/xportal/xmain?xpid=INE&xpgid=ine_publicacoes&PUBLICACOESpub_boui=263714091&PUBLICACOESmodo=2), where the total cholesterol in the Azorean population was higher (69.7%) than that in the mainland average (63.3%). Ferin et al. [40] also observed higher concentrations of total cholesterol, HDL cholesterol, apoA-I, and apoB in an Azorean sample when compared to a Lisboan sample. In addition to genetic predisposition, environmental and lifestyle factors, particularly diet, play a crucial role in shaping cardiometabolic risk. The Azorean population is known to have distinct socio-cultural and dietary patterns, which may influence the observed cardiovascular risk profiles. Traditional dietary habits in the region are typically characterized by a high intake of animal fats, meats, and refined carbohydrates, and a relatively low consumption of fresh fruits, vegetables, and dietary fiber [41]. These patterns may partially explain the higher prevalence of metabolic syndrome and related conditions reported in the Plano Regional de Saúde dos Açores 2030 [31]. While the present study focused on genetic risk, we recognize the importance of considering these environmental influences in future research, ideally through integrated approaches that combine genomic, nutritional, and clinical data. Overall, these data contribute to validating the higher atherosclerotic genetic risk for the Azoreans demonstrated in the present study. Despite that, most high-risk AT-MGPs carry the *APOE* rs7412 variant in the homozygous (CC) state. According to PharmGKB, patients with the rs7412 CC genotype may have decreased response to atorvastatin than patients with the CT or TT genotype (level of evidence 2B). It would be of interest to apply the GRS analysis, as described in the Materials and Methods section (Section 2), to patients undergoing atorvastatin treatment in order to evaluate drug efficacy and the risk of cardiovascular events in the Azorean population, as well as in other populations. According to Madika et al. [42], women had a poorer control of cardiovascular risk with higher systolic blood pressure and LDL-c. In the present study, there is no statistically significant difference between AT risk for female (10.2%) and male (10.6%) groups. However, for VTE, the results indicated a significantly higher risk in women (7.1%) than in men (1.2%; *p* = 0.011; 95% CI: 1.31–38.67). Although this result is only marginally significant after Bonferroni correction (adjusted threshold *p* < 0.0125), it still suggests a potentially relevant sex-based difference that should be explored in future case–control studies with larger sample sizes. Trinchero et al. [43] reported that, among patients with first symptomatic isolated acute deep vein thrombosis (DVT), women had distal DVT more often than men, whereas men had a higher proportion of proximal DVT events. However, a literature search evidenced that men have a higher risk of suffering from thromboembolism than women, regardless of age group [44]. Interestingly, none of the seven female participants from the mainland Portuguese sample were classified as having a high-risk VTE-MGP profile. However, we acknowledge the limited statistical power due to the small number of female participants, which precludes any definitive conclusions. Further studies with larger and more balanced samples are needed to confirm this observation.

Concerning the Azorean geographic distribution, we also observed statistically significant differences. The Eastern group presented the highest VTE risk (8.6%), and no high-risk VTE-MGPs were identified in the Western group. The majority of Eastern individuals with high-risk VTE were women (10 females out of 12 individuals). Conversely, for the risk of AT-MGPs, the Central group was the one with the highest risk (12.9%). These results agree with a previous study performed by our research group [34]. It would be interesting to investigate these differences among Azorean geographic groups within a case–control study.

According to PharmGKB, both factor V Leiden and prothrombin variants are associated with hormonal contraceptives for systemic use. In the present study, 10 females were heterozygous for rs6025 (*F5*:c.1601GA). With a level of evidence 2B, the available information in PharmGKB is that patients with this genotype may have an increased risk of experiencing thrombosis when receiving oral contraceptives as compared to patients with the GG genotype. However, conflicting evidence has been reported, i.e., both factor V Leiden and oral contraceptives have been found to independently increase the risk for thrombosis; but together they may have cumulative effect on VTE risk. At the present time, the standard care when starting contraception consists of identifying the presence of hereditary thrombophilia solely based on the familial history of VTE [45], a strategy that has been reported as poorly predictive of hereditary thrombophilia [46]. An affordable and rational approach could involve the baseline screening of the prothrombotic state to provide objective data supporting the selection of safer contraceptive methods. Taken together, these findings reinforce the need to assess overall VTE risk in women using hormonal contraceptives, within the framework of personalized medicine aimed at reducing the likelihood of thrombotic events.

We evaluated the genetic profiles of each group (AT and VTE); however, to determine whether the two conditions were directly related, we performed a combined analysis of the four SNPs associated with thromboembolism and the 15 SNPs associated with atherosclerosis. In fact, for the Azorean and mainland Portuguese populations, the results showed that the high-risk VTE-MGPs presented a lower risk for atherosclerosis. The same is true for the genetic profiles with a high risk for atherosclerosis. We can infer that, although sharing the same risk factors, the risk of developing an atherosclerotic event is not strongly associated with the risk of developing a VTE event or vice versa. Moreover, the observation that high-risk genetic profiles for atherosclerosis and venous thromboembolism tend to be mutually exclusive highlights the complexity of the genetic architecture underlying these conditions, even when sharing common risk factors such as diabetes, obesity, arterial hypertension, and environmental influences.

## 5. Conclusions

Overall, the data presented here suggest a risk of developing a cardiovascular disease consistent with the European population. However, the Azorean-specific genetic background and socio-cultural habits (dietary and sedentary) may explain the statistically significant differences observed within geographical and sex groups. In conclusion, these findings can be used to improve the prevention, diagnosis, and treatment of high-risk individuals, and contribute to reducing the lifelong burden of cardiovascular diseases in the Azorean population.

## 6. Study Limitations

The present study has some limitations, with the need to validate the results in a case–control study being the most significant. Another constraint is the unavailability, for both samples here studied, of information about other biochemical biomarkers and traditional risk factors, such as smoking and obesity, limiting the interpretation of data. Furthermore, we acknowledge the limitations of a relatively small sample size and the potential underpowering caused by rare variants or subtle effects. However, this initial characterization offers valuable population-level insights and establishes a foundation for future studies with expanded cohorts and case–control designs. Finally, this study confirms the necessity to perform epidemiological studies in the Azorean population, as well as in other Portuguese populations.

## Figures and Tables

**Table 1:** Allele frequencies in the mainland Portuguese and Azorean populations, in comparison with four populations from the gnomAD database.

Gene or Genomic Region	dbSNP ID	Variant Effect *	Allele Frequencies					
			Portugal		gnomAD			
			Mainland	Azores	EUR	AFR	AMR	SAS
*F5*	rs6025	**A**	0.028	0.021	0.022	0.004	0.008	0.014
*F2*	rs1799963	**A**	0.019	0.024	0.013	0.002	0.011	0.002
*MTHFR*	rs1801133	**T**	0.375	0.326	0.337	0.109	0.478	0.149
	rs1801131	**C**	0.309	0.279	0.313	0.163	0.177	0.411
*SORT1*	rs12740374	**T**	0.208	0.185	0.221	0.249	0.210	0.244
*ADAMTS7*	rs3825807	**T**	0.574	0.541	0.446	0.152	0.246	0.348
*PCSK9*	rs11206510	**T**	0.782	0.821	0.182	0.137	0.110	0.049
	rs11591147	T	0.009	0.009	0.017	0.002	0.007	0.001
	rs562556	**A**	0.880	0.821	0.824	0.790	0.904	0.874
	rs505151	**G**	0.037	0.035	0.966	0.736	0.967	0.977
*APOE*	rs405509	**T**	0.644	0.412	0.523	0.747	0.502	0.444
	rs429358	**C**	0.088	0.094	0.151	0.221	0.107	0.101
	rs7412	C	0.894	0.915	0.078	0.106	0.035	0.042
	rs439401	**T**	0.264	0.274	0.630	0.853	0.502	0.442
*LDLR*	rs2228671	T	0.139	0.097	0.123	0.038	0.072	0.071
	rs688	**T**	0.157	0.218	0.446	0.102	0.421	0.393
	rs1433099	T	0.301	0.291	0.731	0.461	0.788	0.673
9p21	rs10757274	**G**	0.389	0.479	0.496	0.217	0.457	0.523
	rs1333049	**C**	0.421	0.453	0.488	0.239	0.483	0.515

Data obtained from the gnomAD database v4.1.0 (https://gnomad.broadinstitute.org/), accessed on 26 August 2024. PT—mainland Portugal; EUR—Europeans (non-Finish); AFR—African/African American; AMR—admixed American; SAS—South Asian. * The risk alleles for atherosclerosis and venous thromboembolism are in bold, and the protective alleles are underlined.

**Table 2:** Haplotype frequencies in the mainland Portuguese and Azorean populations.

Gene or Genomic Region	Haplotypes *					Haplotype Frequencies						
						Mainland Portugal	Azores					
							Total	Geographic Group			Sex	
								Eastern	Central	Western	Male	Female
*MTHFR*	H1	C	A			0.787	0.765	0.814	0.671	0.867	0.718	0.812
	H2	C	**C**			0.102	0.094	0.043	0.171	0.033	0.129	0.059
	H3	**T**	A			0.111	0.141	0.143	0.157	0.100	0.153	0.129
*PCSK9*	H1	C	G	**A**	A	0.190	0.134	0.170	0.132	0.085	0.088	0.170
	H2	C	G	G	A	0.018	0.036	0.071	−	0.015	0.057	0.018
	H3	C	G	G	**G**	−	0.003	0.002	0.004	−	0.009	−
	H4	C	T	G	A	0.004	0.006	0.007	0.007	−	0.006	0.006
	H5	C	G	**A**	**G**	0.001	<0.001	−	−	−	0.005	−
	H6	C	T	**A**	A	0.005	−	−	−	−	−	−
	H7	**T**	G	**A**	A	0.655	0.657	0.584	0.696	0.715	0.683	0.642
	H8	**T**	G	**A**	**G**	0.028	0.026	0.039	0.008	0.033	0.018	0.029
	H9	**T**	G	G	A	0.091	0.128	0.125	0.130	0.151	0.131	0.123
	H10	**T**	G	G	**G**	0.008	0.006	0.002	0.016	−	0.003	0.006
	H11	**T**	T	**A**	A	−	0.003	−	0.007	−	−	0.006
*APOE*	H1	G	**C**	C	C	−	0.028	0.009	0.058	−	−	0.005
	H2	G	**C**	C	**T**	0.038	0.001	0.006	−	−	0.424	0.463
	H3	G	T	C	C	0.485	0.434	0.405	0.420	0.583	0.029	0.041
	H4	G	T	C	**T**	0.014	0.040	0.053	0.036	−	0.076	0.078
	H5	G	T	T	C	0.103	0.085	0.105	0.084	−	−	0.009
	H6	G	T	T	**T**	0.002	<0.001	−	0.002	−	−	−
	H7	G	**C**	T	C	0.001	−	−	−	−	0.047	0.081
	H8	**T**	**C**	C	C	0.049	0.065	0.048	0.092	0.033	0.111	0.117
	H9	**T**	T	C	C	0.060	0.114	0.138	0.103	0.083	0.283	0.177
	H10	**T**	T	C	**T**	0.248	0.233	0.234	0.205	0.267	−	0.004
	H11	**T**	**C**	T	C	−	<0.001	0.002	−	−	−	0.003
	H12	**T**	T	T	**T**	−	<0.001	−	−	0.033	−	0.005
*LDLR*	H1	C	C	C		0.461	0.427	0.407	0.408	0.517	0.429	0.424
	H2	C	C	T		0.261	0.282	0.271	0.321	0.217	0.306	0.258
	H3	C	**T**	C		0.119	0.187	0.228	0.157	0.167	0.153	0.223
	H4	C	**T**	T		0.020	0.007	0.007	0.008	−	−	0.012
	H5	T	C	C		0.100	0.074	0.071	0.079	0.067	0.076	0.071
	H6	T	**T**	C		0.019	0.021	0.007	0.029	0.033	0.029	0.012
	H7	T	**T**	T		−	0.002	0.007	−	−	0.006	−
	H8	T	C	T		0.020	−	−	−	−	−	−
9p21	H1	A	**C**			0.052	0.019	0.039	0.007	−	0.018	0.019
	H2	A	G			0.559	0.502	0.489	0.478	0.583	0.523	0.481
	H3	**G**	**C**			0.369	0.434	0.389	0.486	0.417	0.417	0.452
	H4	**G**	G			0.020	0.045	0.082	0.029	−	0.042	0.048

* The risk alleles for atherosclerosis and venous thromboembolism are in bold, and the protective alleles are underlined.

**Table 3:** Relevant atherosclerosis multilocus genetic profiles (AT-MGPs) of the mainland Portuguese and Azorean populations (high risk: percentile 9; low risk: percentile 1).

AT-MGP ID *	RV ^#^	PV ^$^	HoR ^&^	GRS ^+^	AT-MGP Relative Frequency								
					Mainland Portugal			Azores					
					Total	Sex		Total	Geographic Group			Sex	
						Male	Female		Eastern	Central	Western	Male	Female
**High risk**													
AT-MGP1	15	2	6	16.17	0.009	-	0.143	-	-	-	-	-	-
AT-MGP2	12	3	3	15.20	0.009	0.010	-	-	-	-	-	-	-
AT-MGP3	12	3	3	15.13	-	-	-	0.006	-	0.014	-	0.012	-
AT-MGP4	12	3	4	14.80	-	-	-	0.006	-	-	0.033	0.012	-
AT-MGP5	10	4	3	14.54	-	-	-	0.006	0.014	-	-	0.012	-
AT-MGP6	13	4	5	14.54	-	-	-	0.006	-	0.014		-	0.009
AT-MGP7	13	2	4	14.16	-	-	-	0.006	0.014	-	-	-	0.009
AT-MGP8	13	2	5	13.92	-	-	-	0.006	-	-	0.033	-	0.009
AT-MGP9	13	2	5	13.88	-	-	-	0.006	0.014	-	-	0.012	-
AT-MGP10	13	2	3	13.86	-	-	-	0.006	0.014	-	-	-	0.009
AT-MGP11	14	2	6	13.83	-	-	-	0.006	0.014	-	-	-	0.009
AT-MGP12	12	2	3	13.83	-	-	-	0.006	-	0.014	-	0.012	-
AT-MGP13	12	2	4	13.83	0.009	0.010		-	-	-	-	-	-
AT-MGP14	12	3	3	13.77	-	-		0.006	-	0.014	-	-	0.009
AT-MGP15	12	3	4	13.70	0.009	0.010		-	-	-	-	-	-
AT-MGP16	13	3	5	13.66	0.009	-	0.143	-	-	-	-	-	-
AT-MGP17	12	4	4	13.55	-	-	-	0.006	-	0.014	-	-	0.009
AT-MGP18	11	4	4	13.48	-	-		0.006	-	0.014	-	0.012	-
AT-MGP19	11	4	5	13.38	-	-	-	0.006	0.014	-	-	-	0.009
AT-MGP20	14	1	6	13.19	-	-	-	0.006	-	0.014	-	-	0.009
AT-MGP21	11	2	3	12.86	-	-	-	0.006	0.014	-	-	0.012	-
AT-MGP22	13	2	6	12.82	0.009	0.010	-	-	-	-	-	-	-
AT-MGP23	11	3	3	12.70	0.009	0.010	-	-	-	-	-	-	-
AT-MGP24	12	2	4	12.65	-	-	-	0.006	-	0.014	-	0.012	-
AT-MGP25	11	3	4	12.64	-	-	-	0.006	-	0.014	-	0.012	-
AT-MGP26	12	3	5	12.61	0.009	0.010		-	-	-	-	-	-
AT-MGP27	11	3	5	12.59	-	-	-	0.006	-	-	0.033	-	0.019
				**Total**	**0.074**	**0.059**	**0.286**	**0.112**	**0.100**	**0.129**	**0.100**	**0.106**	**0.102**
**Low Risk**													
AT-MGP28	8	2	4	5.98	-	-	-	0.006	0.0143	-	-	-	0.009
AT-MGP29	6	2	1	5.95	0.009	0.010	-	-	-	-	-	-	-
AT-MGP30	7	2	2	5.89	0.009	0.010	-	-	-	-	-	-	-
AT-MGP31	7	3	2	5.87	-	-	-	0.006	-	0.0143	-	-	0.009
AT-MGP32	7	2	3	5.86	-	-	-	0.006	-	-	0.0333	-	0.009
AT-MGP33	7	2	3	5.86	0.009	0.010	-	-	-	-	-	-	-
AT-MGP34	4	3	0	5.82	-	-	-	0.006	0.0143	-	-	0.0118	-
AT-MGP35	5	3	1	5.75	-	-	-	0.006	-	0.0143	-	0.0118	-
AT-MGP36	5	3	1	5.74	0.009	0.010	-	-	-	-	-	-	-
AT-MGP37	7	3	3	5.68	0.009	0.010	-	-	-	-	-	-	-
AT-MGP38	7	3	3	5.65	-	-	-	0.006	-	-	0.0333	-	0.009
AT-MGP39	6	2	2	5.02	-	-	-	0.006	0.014	-	-	-	0.012
AT-MGP40	7	2	1	4.94	0.009	0.010	-	-	-	-	-	-	-
AT-MGP41	5	2	2	4.90	-	-	-	0.006	-	-	0.033	0.012	-
AT-MGP42	5	3	1	4.79	-	-	-	0.006	0.014	-	-	-	0.012
AT-MGP43	4	3	0	4.76	-	-	-	0.006	0.014	-	-	0.012	-
AT-MGP44	6	2	1	4.92	-	-	-	0.012	0.0143	-	0.0333		0.009
AT-MGP45	7	2	3	4.78	-	-	-	0.006	-	-	0.0333	0.0118	-
AT-MGP46	5	3	1	4.73	-	-	-	0.006	0.0143	-	-	-	0.009
AT-MGP47	5	3	1	4.70	0.009	0.010	-	-	-	-	-	-	-
AT-MGP48	6	3	2	4.61	0.009	0.010	-	-		-	-	-	-
AT-MGP49	7	1	3	4.00	-	-	-	0.006	-	0.0143	-	0.0118	-
AT-MGP50	6	2	2	3.86	0.009	0.010	-	-	-	-	-	-	-
AT-MGP51	5	2	2	3.72	-	-	-	0.006	0.0143	-	-	0.0118	-
AT-MGP52	6	2	2	3.71	0.009	-	0.143	-	-	-	-	-	-
AT-MGP53	3	3	1	3.63	-	-	-	0.006	0.0143	-	-	-	0.009
AT-MGP54	7	0	3	3.26	0.009	0.010	-	-	-	-	-	-	-
				**Total**	**0.120**	**0.119**	**0.143**	**0.088**	**0.100**	**0.057**	**0.133**	**0.071**	**0.074**

* MGPs’ SNP composition is described in Supplementary Table S3. ^#^ RV—no. of risk alleles; ^$^ PV—no. of protective alleles; ^&^ HoR—no. of SNPs with homozygosity for the risk alleles; ^+^ GRS—genetic risk score using a weighted-risk allele count method.

**Table 4:** Relevant venous thromboembolism multilocus genetic profiles (VTE-MGPs) of the mainland Portuguese and Azorean populations (high risk: percentile 9; low risk: percentile 1).

VTE-MGP ID *	RV ^#^	PV ^$^	HoR ^&^	GRS ^+^	VTE-MGP Relative Frequency									
					Mainland Portugal				Azores					
					Total	Sex		Total		Geographic Group			Sex	
						Male	Female			Eastern	Central	Western	Male	Female
**High risk**														
VTE-MGP1	3	0	0	6.18	0.009	0.010	-	-		-	-	-	-	-
VTE-MGP2	1	0	0	4.38	-	0.010	-	0.006		0.014	-	-	-	0.012
VTE-MGP3	2	0	0	4.38	-	-	-	0.024		0.043	0.014		0.012	0.035
VTE-MGP4	2	0	0	4.38	0.009	0.010	-	0.012		0.029	-	-	-	0.024
VTE-MGP5	3	0	1	4.38	0.009	0.010	-	-		-	-	-	-	-
VTE-MGP6	3	0	0	4.38	0.009	0.010	-	-		-	-	-	-	-
VTE-MGP7	3	0	1	4.38	0.009	0.010	-	-		-	-	-	-	-
				**TOTAL**	**0.045**	**0.059**	**0.000**	**0.041**		**0.086**	**0.014**	**0.000**	**0.012**	**0.071**
**Low Risk**														
VTE-MGP8	0	0	0	0.00	0.102	0.099	0.143	0.153		0.114	0.157	0.233	0.153	0.153
VTE-MGP9	1	0	0	0.00	0.139	0.149	-	0.194		0.114	0.257	0.233	0.153	0.235
VTE-MGP10	2	0	1	0.00	0.083	0.089	-	0.088		0.043	0.157	0.033	0.118	0.059
VTE-MGP11	1	0	0	0.00	0.241	0.228	0.429	0.212		0.229	0.171	0.267	0.235	0.188
VTE-MGP12	2	0	0	0.00	0.250	0.257	0.143	0.129		0.186	0.071	0.133	0.129	0.129
VTE-MGP13	2	0	1	0.00	0.093	0.079	0.286	0.141		0.143	0.157	0.100	0.153	0.129
VTE-MGP14	3	0	1	0.00	0.009	0.010	-	-		-	-	-	-	-
				**TOTAL**	**0.824**	**0.911**	**1.000**	**0.918**		**0.829**	**0.971**	**1.000**	**0.941**	**0.894**

* MGPs’ SNP composition is described in Supplementary Table S4. ^#^ RV—no. of risk alleles; ^$^ PV—no. of protective alleles; ^&^ HoR—no. of SNPs with homozygosity for the risk alleles; ^+^ GRS—genetic risk score using a weighted-risk allele count method.

## Data Availability

The data supporting the findings of this study are available from the corresponding author upon reasonable request.

## Supplementary Materials

The following supporting information can be downloaded at: https://www.mdpi.com/article/10.3390/cimb47080625/s1, references [47,48,49,50,51,52,53,54,55,56,57,58,59,60,61,62,63,64,65,66,67,68] are cited in Supplementary File.
